# Things Able to Treat Pain

**DOI:** 10.3390/ijms241210346

**Published:** 2023-06-19

**Authors:** Anna Maria Aloisi

**Affiliations:** Department of Medicine, Surgery and Neuroscience, University of Siena, 53100 Siena, Italy; annamaria.aloisi@unisi.it

Chronic pain is a medical condition that affects a considerable number of people of all ages. It can affect all organs of the body, including the CNS. Neuropathic pain has several origins and is particularly difficult to treat since neural pathways of different origins are involved in peculiar anatomical contexts. A number of attempts have been made to treat it, but at present, most patients are unsatisfied with the proposed therapies, and most of the time the treatments are without real benefits and have side effects. Due to the long-term treatment or lifelong duration of many cases, drugs often enhance the painful condition.

In this Special Issue, the various studies presented all indicate a desire to understand chronic pain mechanisms while also suggesting modulatory substances that are often far from those classically considered analgesics. An interesting example is the study by Karadi et al. [1], investigating the analgesic effect of renin-angiotensin system (RAS) antagonists. They have already shown the connection between the RAS and neuropathic pain (NP) and the possible pharmacological interactions between the opioid and angiotensin systems [2]. In the present study, the authors tested the effect of angiotensin II receptor blockers (ARB) with morphine in a rat neuropathic pain model. This combination increased the analgesic effect of morphine alone and, importantly, the ARB telmisartan reduced the development of opioid analgesic tolerance during morphine treatment of chronic pain.

Another substance that appears to be able to modulate the pain/analgesia mechanism is ghrelin [3]. It is secreted by the stomach into the blood, and its receptors have been found in several CNS areas also involved in pain modulation and/or transmission. Ghrelin has been shown to have an inhibitory effect on inflammatory pain through interference with the central opioid system [4]. Indeed, it has stimulating effects on neurons in the arcuate nucleus, which contains endogenous opioid-containing neurons. Pirzadeth et al. [5] describe the CNS administration of ghrelin in a model of persistent pain (formalin test) in rats. They found an analgesic effect, confirming the interaction between ghrelin and the opioid system.

Luu et al. [6] focus on an ATP-sensitive K+ channel known to be involved in hypersensitivity during chronic pain. They describe different KATP channel subunits possibly involved in neuropathic pain in mice. In a chronic pain model (spinal nerve ligation), SUR1 and Kir6.2 subunits were found to be downregulated in the dorsal root ganglia and spinal cord, a condition that can exacerbate neuropathic pain symptoms. Potassium channels can be considered a target for analgesic therapies because they are expressed in nociceptors and could play a role in regulating the excitability of neurons involved in pain transmission [7]. In fact, intraplantar and intrathecal administration of SUR-1 subtype agonists helped to alleviate mechanical hypersensitivity and improve motility.

Severyanova et al. [8] describe the effects of L-lysine on cellular physiological processes. Lysine is an essential acid incorporated into almost all proteins in animals and plants. It has several actions, such as cell proliferation, differentiation, and excitability, as well as intercellular interaction by means of the neurotransmitter system [9]. It was shown that a deficiency of L-lysine in rats resulted in a decreased release of 5HT in the central nucleus of the amygdala and NA in the VM hypothalamus, a condition that could be counteracted by reuptake inhibitors. The results showed the importance of L-lysine in the biological significance of pain, particularly in behavior.

Li et al. [10] describe the action of serotonin–noradrenaline reuptake inhibitors (SNRI) in a model of neuropathic pain, with particular attention to the role of oxaliplatin. The SNRI are involved in endogenous analgesic mechanisms. In this rat model of neuropathic pain, venlafaxine had a potent effect. Moreover, the involvement of both alpha-2 adrenergic and 5HT3 receptors in cold and mechanical allodynia, respectively, was shown.

Guindon et al. [11] used a model of HIV-associated neuropathic pain in both male and female mice. The involvement of the viral protein Gp120 in the pathogenesis of HIV-related neuropathic pain has been demonstrated in animal models. Women represent a greater proportion of chronic pain patients. In the present experiment, female mice showed a lower pain threshold than males, and ovariectomy appeared to reduce this sensitivity.

The review by Zajaczkowska et al. [12] focuses on chemotherapy-induced peripheral neuropathy (CIPN), a predominantly sensory neuropathy that may be accompanied by motor and autonomic changes. CIPN is significant in the lives of patients and clinicians because it affects a large number of patients and can last for several years. The authors of this review summarize the known pathogenetic mechanisms through which chemotherapeutic agents frequently induce peripheral neuropathies. The authors describe several animal studies aimed at understanding possible remedies, particularly regarding substances with a wide range of activities to help with chronic pain. However, fewer studies concern possible drugs in research on humans, and in this case, the data are less clear.

Overall, these studies confirm the presence of several pathways in the CNS that could be modulated to obtain pain relief. It is interesting to imagine that these systems are active/inactive in several physiological conditions, which would suggest unexpected modulation, such as by food in the stomach.
